# Evaluation of Turkish dentists’ knowledge about oral cancer and oral mucosal lesions

**DOI:** 10.1186/s12903-024-04533-x

**Published:** 2024-06-29

**Authors:** Zeynep Gümrükçü, Mert Karabağ

**Affiliations:** Department of Oral and Maxillofacial Surgery, Faculty of Dentistry, Recep Tayyip Erdoğan University, Rize, Turkey

**Keywords:** Oral cancer, Questionnaire, Oral mucosal lesions, Dentists, Knowledge

## Abstract

**Background/purpose:**

Oral cancer, including malignancies of the tongue, lips, floor of the mouth, cheek mucosa, gums, palate, and oropharynx, is life-threatening. Early diagnosis and appropriate treatment are crucial for long-term survival. Dentists frequently encounter oral cancers due to the nature of their work. This study aimed to evaluate the knowledge and experience of dentists in Turkey regarding oral cancers.

**Materials and methods:**

A total of 361 participants were included in the study, and survey questions were sent via email. The survey consisted of 16 questions measuring demographic data and knowledge about oral cancerous lesions. Participants were grouped based on their specialization and knowledge level, and differences in responses among groups were examined.

**Results:**

Only 21.3% of the participants felt they had sufficient knowledge and experience about oral cancerous lesions. Overall, the correct answer rates indicated a moderate level of knowledge and experience. When grouped by specialization, oral surgeons had the highest accuracy in their responses (*p* < 0.05).

**Conclusion:**

Dentists are the professional group that most frequently encounters clinically oral cancerous lesions. Therefore, it is critically important for them to be knowledgeable and experienced to reduce morbidity and mortality through early diagnosis. This study evaluated the knowledge status of dentists in Turkey regarding oral cancer and highlighted the need for improved education.

**Supplementary Information:**

The online version contains supplementary material available at 10.1186/s12903-024-04533-x.

## Introduction

Oral cancer, defined as malignancies of the tongue, lips, floor of the mouth, cheek mucosa, gingiva, palate, and oropharynx, is a life-threatening condition [1]. In 2024, a total of 58,450 new oropharyngeal cancer cases and 12,230 deaths due to oropharynx cancers are expected in the United States alone [2]. In a study examining epidemiological data on 36 types of cancer collected from 185 countries, it was reported that there were a total of 389,485 new cases of lip/oral cavity cancer and 188,230 deaths due to these cancers in 2022 [3]. Although studies show that survival times for oropharynx cancers are increasing, the increase in the incidence of cancer is cause for concern [4]. Tobacco use, alcohol consumption, and exposure to ultraviolet (UV) radiation are the most commonly reported risk factors for oral cancer in the literature [5, 6]. Additionally, it is known that human papillomavirus (HPV) infection also plays a role in increasing the risk of oropharyngeal cancer [7].

Early diagnosis of oral premalignant/malignant lesions and appropriate referral of patients can significantly influence the prognosis [8–10]. Studies have shown that the survival rate of late-stage oral lesions is 51%, whereas the survival rate of lesions diagnosed in the early stage can rise up to 84% [9, 11]. According to the health services of the Ministry of Health of the Republic of Turkey, while there were 675 cases of lip-oral space cavity diagnosed in 2014, this number increased to 837 in 2018 [12]. Also according to data from the Global Cancer Observatory (GLOBOCAN), 2246 new lip-oral cavity cases were encountered in Turkey in 2022 and 551 deaths occurred due to oral cancers. This high mortality rate reveals the importance of early diagnosis [3]. Early diagnosis of oral cancers is directly related to the knowledge and experience of dentists on the subject [11]. Many countries such as the United States of America, Spain, Italy, and Canada have conducted studies on dentists' awareness of oral cancer [13–16].

Studies conducted in Turkey regarding the prevalence and prognosis of oral cancer show that squamous cell carcinomas are the most common cancers in the oral cavity following laryngeal carcinoma among head and neck cancers [17, 18]. Therefore, the awareness of Turkish dentists about oral cancer becomes crucial [19, 20]. This study aimed to examine the awareness of Turkish dentists about the diagnosis and treatment process of oral premalignant/malignant lesions.

## Materials and methods

The study was initiated with the approval of the Recep Tayyip Erdoğan University Non-Interventional Ethics Committee (Date: 16.12.2019, Approval number 2019/200). The study was conducted cross-sectionally on 361 dentists who were actively practicing in Turkey between January 2020 and January 2023. Survey questions were sent via e-mail to all dentists registered with the Turkish Dental Association (actively practicing their profession), regardless of age, field of expertise or title. The survey questions were sent to the participants via email, and the data belonging to the participants were obtained through emails. Participants were selected on a voluntary basis, and it was stated that all participants’ data would be recorded anonymously. The survey consisted of 16 questions, 15 of which were multiple-choice (Table 1)(See in the end of manuscript). While determining the content of the survey questions, previous studies in the literature similar to ours were examined and these studies were used [13–16, 19, 21–25]. In addition, the contents of the Turkish dentistry education curriculum were taken as a basis in the selection of questions and answers that questioned objective information.

**Table 1:**
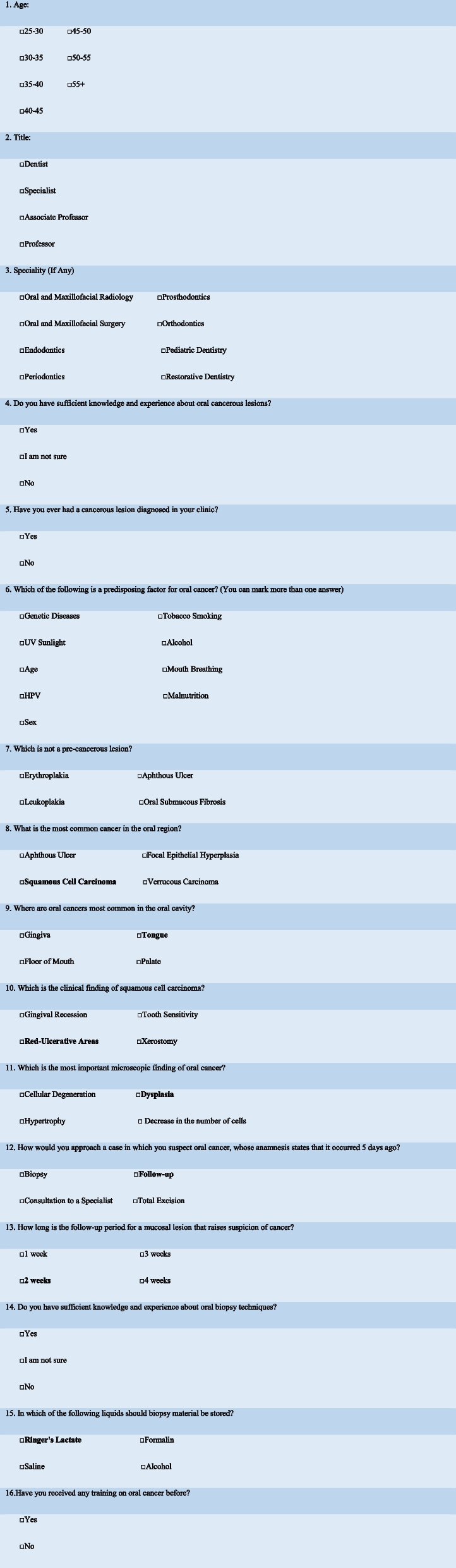
**Survey Questions**

The first three questions of the survey were prepared to collect demographic data such as age, title, and the specialty field of the participants. The fourth and fifth questions were aimed at subjectively determining the participants’ experiences with oral cancer. The subsequent 10 questions inquired about objective information regarding oral premalignant and malignant lesions. The last question of the survey asked whether the participants had received any specific training on oral cancers. The correct answers to the questions of the survey asking objective information about oral cancers are marked in bold in Table 1. (Questions 8, 9, 10, 11, 12, 13, 15).

Participants were divided into separate groups according to their answers to the 3rd, 4th and 5th questions of the survey, and statistical analysis was performed to determine whether there was a statistically significant difference between the answers given by the formed groups. Participants were grouped according to specialty and examined the effect of dentists’ professional orientation on awareness about oral cancer. The participants were divided into groups according to their answers to the survey question inquiring about their knowledge and experiences about oral cancers (Yes/Not Sure/No). The reason for this was to examine the counterpart of the participants’ subjectively stated knowledge and experience in professional practice. Participants were examined in two different subgroups according to whether they had previously been clinically diagnosed with a cancerous lesion, thus aiming to examine the effect of clinical experience on oral cancer awareness.

In calculating the sample size of the study, power (test power) was determined to be at least 80% and Type-1 error at 5% for each variable. The normality of continuous measurements in the study was examined using Shapiro–Wilk and Skewness-Kurtosis tests, and parametric tests were used because the measurements were normally distributed. Descriptive statistics for continuous variables in the study are expressed as number (*n*) and percentage (%). The relationships between “categorical (personal) factors” and “responses to questions” were determined using the Chi-square and Fisher’s exact tests. A statistical significance level of *p* < 0.05 was adopted for calculations, and the IBM SPSS for Windows, ver.26 statistical package program was used for analyses.

## Results

The demographic data of the participants are presented in Table 2. Upon examination of these data, it was observed that the majority of participants (79.8%) fell within the age range of 25–35 years. Additionally, it was determined that 82.5% (*n* = 298) of the participants were general dentists (GD), followed by oral and maxillofacial surgeons (8.3%/*n* = 30). When asked “Do you have sufficient knowledge and experience about oral cancerous lesions?” Some 62.3% of the participants responded with “Not sure” while 21.3% responded with “Yes”. In response to the fifth question of the survey, “Have you ever diagnosed a cancerous lesion in your clinic before?”, it was found that 33% of the participants had previously diagnosed a cancerous lesion (Table 3).

**Table 2:** Demographic data of the participants

		***n***	**%**
Age	25–35	288	79.8%
	36–45	38	10.5%
	46–55	20	5.5%
	55 +	15	4.2%
Title	Assistant professor	20	5.5%
	Dentist	298	82.5%
	Professor	2	0.6%
	Specialist dentist	41	11.4%
Specialty	Oral and Maxillofacial Surgery	30	8.3%
	Endodontics	7	1.9%
	Oral Diagnosis and Radiology	7	1.9%
	Orthodontics	3	0.8%
	Pedodontics	8	2.2%
	Periodontology	11	3.0%
	Prosthodontics	20	5.5%
	Restorative Dentistry	3	0.8%
	None	272	75.3%

**Table 3:** Responses to Questions 4 and 5 of the survey

Do you have sufficient knowledge and experience about oral cancerous lesions?	Not sure	225	62.3%
	Yes	77	21.3%
	No	59	16.3%
Have you ever had a cancerous lesion diagnosed in your clinic?	Yes	119	33.0%
	No	242	67.0%

In the question querying the predisposing factors of oral cancers, the predisposing factor most selected by participants (99.7%) was “Smoking”. The least marked option, “Mouth Breathing”, was marked at a rate of 21.6%. “Age”- which is not an independent risk factor, the incidence of cancer increases with aging- was marked at a rate of 64.5%. In response to the question, “Which one is not a precancerous lesion?”, 63.7% of the participants gave the correct answer “Aphthous Ulcer”. Following this answer, the most frequent response (22.4%) was “Oral Submucous Fibrosis”. Participants answered “Squamous Cell Carcinoma” as the most common type of cancer in the oral cavity at a rate of 89.5%. The participants marked “Floor of the Mouth” as the most common location for oral cancers at a rate of 45.2%; the correct answer, “Tongue”, was marked at a rate of 36% (Table 6). Participants responded with “Red-ulcerated Areas” the question about the clinical manifestation of squamous cell carcinoma at a rate of 97.5%. In response to the question, “Which is the most important microscopic finding of oral cancer?”, participants answered “Dysplasia” at a rate of 59.3%. Following this answer, the most common response (26.3%) was “Cellular Degeneration”. When asked about the clinical approach to a lesion that appeared 5 days ago, 46.5% of participants chose “Monitor the lesion”, and 45.4% chose “Refer to a specialist”. Participants responded with “2 weeks” to the question about the follow-up period for a lesion suspected of oral cancer at a rate of 60.4%. Participants answered “Formalin” at a rate of 71.7% to the question “In which fluid should biopsy material be stored?” Additionally, 18% of participants stated that they had sufficient knowledge and experience about oral cancers. When asked “Have you received any specific training on oral cancers before?”, 55.7% of participants answered “No” (Table 4).

**Table 4:** Responses to Survey Questions

		***n***	**%**
Which of the following is a predisposing factor to oral cancer?	Genetic Diseases	291	80.6%
	UV Sunlight	262	72.6%
	Age	233	64.5%
	HPV	318	88.1%
	Alcohol	300	83.1%
	Smoking	360	99.7%
	Gender	92	25.5%
	Malnutrition	180	49.9%
	Mouth Breathing	78	21.6%
Which is not a precancerous lesion?	Aphthous Ulcer	230	63.7%
	Erythroplakia	19	5.3%
	Leukoplakia	31	8.6%
	Oral Submucous Fibrosis	81	22.4%
What is the most common cancer in the oral region?	Aphthous Ulcer	18	5.0%
	Focal Epithelial Hyperplasia	11	3.0%
	Squamous Cell Carcinoma	323	89.5%
	Verrucous Carcinoma	9	2.5%
Where are oral cancers most common in the mouth?	Mouth Floor	163	45.2%
	Palate	34	9.4%
	Tongue	130	36.0%
	Gingiva	34	9,4%
Which is the clinical finding of squamous cell carcinoma?	Dry Mouth	4	1,1%
	Tooth Sensitivity	3	0,8%
	Gingival Recession	2	0,6%
	Red-Ulcerative Areas	352	97,5%
Which is the most important microscopic finding of oral cancer?	Dysplasia	214	59,3%
	Hypertrophy	45	12,5%
	Decrease in the number of cells	7	1,9%
	Cellular Degeneration	95	26,3%
How would you approach a case in which you suspect oral cancer, which is stated to have occurred 5 days ago in your anamnesis?	Biopsy	25	6,9%
	Excision of the Mass	4	1,1%
	Follow-up	168	46,5%
	Referral to a Specialist	164	45,4%
How long is the follow-up period for a mucosal lesion that raises suspicion of cancer?	1 Week	46	12,7%
	2 Weeks	218	60,4%
	3 Weeks	41	11,4%
	4 Weeks	56	15,5%
Do you have sufficient knowledge and experience about oral biopsy techniques?	Not sure	161	44,6%
	Yes	65	18,0%
	No	135	37,4%
In which of the following liquids should biopsy material be stored?	Alcohol	6	1,7%
	Formalin	259	71,7%
	Ringer’s Lactate	61	16,9%
	Saline	35	9,7%
Have you received any training on oral cancer before?	Yes	160	44,3%
	No	201	55,7%

When the relationship between the answers to the survey questions and the participants’ specialties was examined, it was found that the specialty field with the highest rate of “Yes” responses to the question “Do you have sufficient knowledge and experience about oral cancerous lesions?” was Oral and Maxillofacial Surgery (23.4%). Similarly, the specialty field with the highest rate (23.4%) of “Yes” responses to the question, “Have you ever diagnosed a cancerous lesion in your clinic before?”, was also Oral and Maxillofacial Surgery, and the specialty field with the highest rate (6.6%) of “No” responses was Prosthetic Dentistry. When asked about the approach to a case suspected of oral cancer with a history of onset 5 days ago, the group that most frequently (12.5%) chose the correct answer “Monitor” was Oral and Maxillofacial Surgery. Conversely, the incorrect answers “Excision of the Mass” (25%) and “Take a Biopsy” (28%) were also most frequently chosen by Oral and Maxillofacial Surgeons. When asked “Do you have sufficient knowledge and experience about oral biopsy techniques?”, the specialty field with the highest rate (32.3%) of “Yes” responses was Oral and Maxillofacial Surgery, and the specialty field with the highest rate (10.4%) of “No” responses was Prosthetic Dentistry. Participants who gave the correct answer to the question, “In which fluid should biopsy material be stored?” at the highest rate (11.6%) belonged to Oral and Maxillofacial Surgery. Lastly, the group with the highest rate (11.9%) of “Yes” responses to the question, “Have you received any specific training on oral cancers before?”, was Oral and Maxillofacial Surgery (Table 5).

**Table 5:** Relationship between Responses and Specialty

		Specialty																		
		**Oral and Maxillo-facial Surgery**		**Endo-dontics**		**Oral Diagnosis and Radiology**		**Ortho-dontics**		**Pedo-dontics**		**Perio-dontology**		**Prostho-dontics**		**Restorative Dentistry**		**None**		*p
		***n***	**%**	***n***	**%**	***n***	**%**	***n***	**%**	***n***	**%**	***n***	**%**	***n***	**%**	***n***	**%**	***n***	**%**	
Do you have sufficient knowledge and experience about oral cancerous lesions?	Not sure	12	2.7%	1	0.4%	2	0.9%	5	2.2%	9	4.0%	12	5.3%	2	0.9%	176	2.7%	1	0.4%	***.001***
	Yes	18	1.3%	6	7.8%	1	1.3%	2	2.6%	0	0.0%	3	3.9%	0	0.0%	46	1.3%	6	7.8%	
	No	0	0.0%	0	0.0%	0	0.0%	1	1.7%	2	3.4%	5	8.5%	1	1.7%	50	0.0%	0	0.0%	
Have you ever had a cancerous lesion diagnosed in your clinic?	Yes	24	0.0%	7	5.9%	1	0.8%	0	0.0%	3	2.5%	4	3.4%	1	0.8%	79	0.0%	7	5.9%	***.001***
	No	6	2.9%	0	0.0%	2	0.8%	8	3.3%	8	3.3%	16	6.6%	2	0.8%	193	2.9%	0	0.0%	
How would you approach a case in which you suspect oral cancer, which is stated to have occurred 5 days ago in your anamnesis?	Biopsy	7	0.0%	0	0.0%	0	0.0%	0	0.0%	1	4.0%	1	4.0%	1	4.0%	15	0.0%	0	0.0%	***.001***
	Excision of the Mass	1	0.0%	0	0.0%	0	0.0%	0	0.0%	0	0.0%	0	0.0%	0	0.0%	3	0.0%	0	0.0%	
	Follow-up	21	1.2%	7	4.2%	0	0.0%	4	2.4%	6	3.6%	7	4.2%	0	0.0%	121	1.2%	7	4.2%	
	Referral to a Specialist	1	3.0%	0	0.0%	3	1.8%	4	2.4%	4	2.4%	12	7.3%	2	1.2%	133	3.0%	0	0.0%	
Do you have sufficient knowledge and experience about oral biopsy techniques?	Not sure	9	0.6%	1	0.6%	1	0.6%	3	1.9%	10	6.2%	3	1.9%	0	0.0%	133	0.6%	1	0.6%	***.001***
	Yes	21	1.5%	4	6.2%	1	1.5%	1	1.5%	1	1.5%	3	4.6%	0	0.0%	33	1.5%	4	6.2%	
	No	0	3.7%	2	1.5%	1	0.7%	4	3.0%	0	0.0%	14	10.4%	3	2.2%	106	3.7%	2	1.5%	
In which of the following liquids should biopsy material be stored?	Alcohol	0	0.0%	1	16.7%	0	0.0%	0	0.0%	0	0.0%	0	0.0%	0	0.0%	5	0.0%	1	16.7%	***.026***
	Formalin	30	1.9%	6	2.3%	3	1.2%	4	1.5%	11	4.2%	15	5.8%	2	0.8%	183	1.9%	6	2.3%	
	Ringer’s Lactate	0	3.3%	0	0.0%	0	0.0%	4	6.6%	0	0.0%	4	6.6%	1	1.6%	50	3.3%	0	0.0%	
	Saline	0	0.0%	0	0.0%	0	0.0%	0	0.0%	0	0.0%	1	2.9%	0	0.0%	34	0.0%	0	0.0%	
Have you received any training on oral cancer before?	Yes	19	1.3%	7	4.4%	3	1.9%	4	2.5%	3	1.9%	10	6.3%	2	1.3%	110	1.3%	7	4.4%	***.006***
	No	11	2.5%	0	0.0%	0	0.0%	4	2.0%	8	4.0%	10	5.0%	1	0.5%	162	2.5%	0	0.0%	

When examining the relationship between participants’ responses to the question, “Do you have sufficient knowledge and experience about oral cancerous lesions?” and their responses to other questions, it was observed that 3.4% of the dentists who had previously diagnosed a cancerous lesion responded “No” (*p* = 0.001). Similarly, among those who answered the question, “Which one is not a pre-cancerous lesion?” correctly, 13.5% responded “No” to having sufficient knowledge and experience, and the majority of participants (60–67.9%) who answered incorrectly chose the option “Not Sure” (*p* = 0.015). Among participants who correctly answered the question, “Which cancer is most commonly seen in the oral region?”, 23.5% stated that they had sufficient knowledge and experience, and this rate was 0–11.1% among those who answered incorrectly (*p* = 0.001). For the question, “Which one is a clinical manifestation of squamous cell carcinoma?”, 21.9% of participants who answered correctly stated that they had sufficient knowledge and experience, and this rate was 0% among those who answered incorrectly (*p* = 0.001). Regarding the question, “What is the most important microscopic finding of oral cancer?”, 28% of participants who answered correctly stated that they had sufficient knowledge and experience, and among those who answered incorrectly, the rate of stating sufficient knowledge and experience was 6.7–14.3% (*p* = 0.005). For the question, “How would you approach a case suspected of oral cancer with a history of onset 5 days ago?”, 31.5% of participants who answered correctly stated that they had sufficient knowledge and experience. In contrast, among those who gave one of the incorrect answers, stating “Take a Biopsy”, 40% stated that they had sufficient knowledge and experience (*p* = 0.001). Regarding the question “What is the follow-up period for a mucosal lesion that raises suspicion of cancer?”, 26.6% of participants who answered correctly stated that they had sufficient knowledge and experience. Among those who answered incorrectly, the rate of stating sufficient knowledge and experience was 8.9–17.1% (*p* = 0.015). Similarly, for the question, “Do you have sufficient knowledge and experience about oral biopsy techniques?”, 75.4% of participants who answered “Yes” stated that they had sufficient knowledge and experience (*p* = 0.001). For the question, “In which of the following fluids should biopsy material be stored?”, 27.4% of participants who answered correctly stated that they had sufficient knowledge and experience, and among those who answered incorrectly, this rate was 3.3–16.7% (*p* = 0.002). When asked “Have you received any training on oral cancer before?”, 34.4% of participants who answered “Yes” stated that they had sufficient knowledge and experience, whereas the rate was 10.9% among those who answered “No” (*p* = 0.001) (Table 6).

**Table 6:** Relationship between answers and knowledge and experience

		Do you have sufficient knowledge and experience about oral cancerous lesions?							
		**Not Sure**		**Yes**			**No**		*p
		***n***	**%**	***n***	**%**	***n***		**%**	
Have you ever had a cancerous lesion diagnosed in your clinic?	Yes	62	52.1%	53	44.5%	4		3.4%	***.001***
	No	163	67.4%	24	9.9%	55		22.7%	
Which is not a precancerous lesion?	Aphthous Ulcer	138	60.0%	61	26.5%	31		13.5%	***.015***
	Erythroplakia	13	68.4%	2	10.5%	4		21.1%	
	Leukoplakia	19	61.3%	2	6.5%	10		32.3%	
	Oral Submucous Fibrosis	55	67.9%	12	14.8%	14		17.3%	
What is the most common cancer in the oral region?	Aphthous Ulcer	16	88.9%	0	0.0%	2		11.1%	***.001***
	Focal Epithelial Hyperplasia	5	45.5%	0	0.0%	6		54.5%	
	Squamous Cell Carcinoma	199	61.6%	76	23.5%	48		14.9%	
	Verrucous Carcinoma	5	55.6%	1	11.1%	3		33.3%	
Which is the clinical finding of squamous cell carcinoma?	Dry Mouth	1	25.0%	0	0.0%	3		75.0%	***.001***
	Tooth Sensitivity	2	66.7%	0	0.0%	1		33.3%	
	Gingival Recession	0	0.0%	0	0.0%	2		100.0%	
	Red-Ulcerative Areas	222	63.1%	77	21.9%	53		15.1%	
Which is the most important microscopic finding of oral cancer?	Dysplasia	128	59.8%	60	28.0%	26		12.1%	***.005***
	Hypertrophy	32	71.1%	3	6.7%	10		22.2%	
	Decrease in the number of cells	5	71.4%	1	14.3%	1		14.3%	
	Cellular Degeneration	60	63.2%	13	13.7%	22		23.2%	
How would you approach a case in which you suspect oral cancer, which is stated to have occurred 5 days ago in your anamnesis?	Biopsy	14	56.0%	10	40.0%	1		4.0%	***.001***
	Excision of the Mass	3	75.0%	1	25.0%	0		0.0%	
	Follow-up	96	57.1%	53	31.5%	19		11.3%	
	Referral to a Specialist	112	68.3%	13	7.9%	39		23.8%	
How long is the follow-up period for a mucosal lesion that raises suspicion of cancer?	1 Week	30	65.2%	7	15.2%	9		19.6%	***.015***
	2 Weeks	133	61.0%	58	26.6%	27		12.4%	
	3 Weeks	27	65.9%	7	17.1%	7		17.1%	
	4 Weeks	35	62.5%	5	8.9%	16		28.6%	
Do you have sufficient knowledge and experience about oral biopsy techniques?	Not sure	133	82.6%	19	11.8%	9		5.6%	***.001***
	Yes	14	21.5%	49	75.4%	2		3.1%	
	No	78	57.8%	9	6.7%	48		35.6%	
In which of the following liquids should biopsy material be stored?	Alcohol	4	66.7%	1	16.7%	1		16.7%	***.002***
	Formalin	150	57.9%	71	27.4%	38		14.7%	
	Ringer’s Lactate	47	77.0%	2	3.3%	12		19.7%	
	Saline	24	68.6%	3	8.6%	8		22.9%	
Have you received any training on oral cancer before?	Yes	90	56.3%	55	34.4%	15		9.4%	***.001***
	No	135	67.2%	22	10.9%	44		21.9%	

When the relationship between the answers to the question, “Have you ever diagnosed a cancerous lesion in your clinic before?” and the answers to other questions was examined, it was found that 39.3% of participants who gave the correct answer to the question about the approach to a case suspected of oral cancer with a history of onset 5 days ago had previously diagnosed a cancerous lesion. However, among those who chose the incorrect answer, “Take a Biopsy”, this rate was 44% (*p* = 0.026). When asked “Do you have sufficient knowledge and experience about oral biopsy techniques?”, 70.8% of participants who answered “Yes" had previously diagnosed a cancerous lesion, whereas among those who answered "Not sure" and "No”, this rate was 27.3% and 21.5%, respectively (*p* = 0.001). The rate of having diagnosed a cancerous lesion was 42.5% among participants who had previously received training on oral cancers, and among those who had not received any training, this rate was 25.4% (*p* = 0.001) (Table 7).

**Table 7:** Relationship between Responses and previous diagnosis status

		**Have you ever had a cancerous lesion diagnosed in your clinic?**				
		**Yes**		**No**		*p
		***n***	**%**	***n***	**%**	
How would you approach a case in which you suspect oral cancer, which is stated to have occurred 5 days ago in your anamnesis?	Biopsy	11	44.0%	14	56.0%	***.026***
	Excision of the Mass	1	25.0%	3	75.0%	
	Follow-up	66	39.3%	102	60.7%	
	Referral to a Specialist	41	25.0%	123	75.0%	
Do you have sufficient knowledge and experience about oral biopsy techniques?	Not sure	44	27.3%	117	72.7%	***.001***
	Yes	46	70.8%	19	29.2%	
	No	29	21.5%	106	78.5%	
Have you received any training on oral cancer before?	Yes	68	42.5%	92	57.5%	***.001***
	No	51	25.4%	150	74.6%	

## Discussion

Dentists are the medical professional group that most frequently encounters oral cancerous lesions in clinical settings [26]. Therefore, it is critically important for dentists to be knowledgeable and experienced in this matter to decrease morbidity/mortality rates through early diagnosis [13]. Hence, our study aimed to evaluate the knowledge and experiences of dentists in Turkey regarding oral cancers.

Of the 361 participants included in the study, 79.8% (*n* = 288) were aged 25–35 years. This high proportion of young participants becomes more explanatory considering that the data were obtained via email. The majority of participants (75.3%) were GDs, the lower percentage of specialist dentists can generally be explained by the low ratio of such dentists in Turkey. Around one-fifth (21.3%) of participants considered their knowledge of oral cancers to be sufficient, 16.3% reported their knowledge as insufficient, and 62.3% were unsure of their knowledge. In a similar study conducted by Kumar et al. in India, the majority of participants reported having sufficient knowledge and experience [27]. However, studies conducted in Yemen and Sudan showed, similar to our study, that the majority of participants did not have sufficient knowledge and experience regarding oral cancers [21, 22]. This difference can be explained by variations in dental education across countries.

In their study, Ojha et al. reported that 59 out of 216 participants frequently encountered oral cancerous lesions. Our study showed that 67% of participants had not diagnosed any cancerous lesions previously, which is parallel to Ojha et al.’s findings [28]. A similar study conducted in Italy revealed that 94.1% of dentists considered smoking and 79.2% considered alcohol consumption as predisposing factors for oral cancerous lesions [16]. Similarly, in our study, most participants identified smoking (99.7%) and alcohol consumption (83.1%) as predisposing factors for oral cancer. The majority of participants in our study also identified ultraviolet sunlight (72.6%) and viral infections (e.g., HPV) (81.1%) as predisposing factors for oral cancers. These results are consistent with many similar studies [22, 29]. Decuseara et al. reported that 55% of dentists considered aging as a risk factor for oral cancer [23]. Although aging alone is not considered a predisposing factor for oral cancers, participants in our study, similar to Decuseara et al., indicated that advanced age was a predisposing factor for oral cancers at a rate of 64.5%. This rate is higher among dentists in Turkey compared with similar studies conducted in other countries. It is known that oral cancers are most commonly seen on the tongue within the oral cavity [24]. In our study, 45.2% of participants responded “floor of mouth” to the question questioning this knowledge, but the correct answer “tongue” was marked by only 36% of participants. Similar to our study, dentists in Iran, Kuwait, and Yemen identified the tongue and floor of mouth as the areas where oral cancers were commonly seen [22, 25, 29]. In our study, 63.7% of participants marked the correct answer to the question about their knowledge of oral precancerous lesions. Different studies have also shown that participants’ knowledge of oral precancerous lesions (e.g., erythroplakia, leukoplakia) is at an acceptable level [16, 21]. In our study, 97.5% of participants provided the correct answer to the question about the clinical manifestations of oral cancer. Kumar et al. reported that participants in India indicated the presence of red-ulcerative areas as an early clinical manifestation of oral cancers at a rate of 9.6%, and Clovis et al. reported that dentists in their study highly associated red-ulcerative lesions with oral cancer [14, 27]. This difference may be explained by variations in dental education between countries and, additionally, by the increased incidence of leukoplakia due to the common betel nut chewing habit in India. In our study, participants described squamous cell carcinoma as the most commonly seen cancerous lesion in the oral region (89.5%). Our study is consistent with other studies in this regard [22, 25]. In a question in our study querying the approach to be followed for a lesion suspected of oral cancer, 45.4% of participants chose the option of referral to a specialist dentist. Similar studies indicate that the approach of dentists to lesions suspected of oral cancer is to refer them to a specialist dentist [22, 27, 30]. Participants in our study were questioned about whether they considered their knowledge of oral cancers to be sufficient, and only 21.3% of participants responded “Yes” to this question. Similar to our study, the majority of participants in a study by Alaizari et al. reported that they perceived their knowledge of oral cancers as insufficient [22].

In the survey investigating predisposing factors of oral cancers, it was observed that incorrect answers such as mouth breathing (21.6%) and genetic disorders (80.6%) were given to question 6. In question 7, which inquired about oral pre-cancerous lesions, 36.3% of the participants marked incorrect answers. Similarly, in the question about the clinical findings of oral cancers, incorrect responses such as dry mouth and tooth sensitivity were selected. Additionally, in question 15 of the survey, which queried the fluid in which biopsy material should be preserved, 16.9% of the participants provided the answer Ringer’s lactate. These incorrect responses highlight the knowledge gaps among Turkish dentists regarding the diagnosis and treatment processes of oral cancerous lesions.

In our study, participants were grouped differently based on their areas of expertise, their declared knowledge and experience regarding oral cancers, and their previous clinical diagnosis of cancerous lesions. Thus, statistical comparisons were made between the groups based on the responses given.

In the statistical analysis conducted among the areas of expertise, the group of oral, dental, and maxillofacial surgeons had the highest rate of correct responses in terms of knowledge, experience, and clinical practices. Similarly, Coella et al. reported in a similar study conducted in Italy that oral surgeons provided a higher rate of correct answers to questions about cancerous lesions compared with other groups [16]. Considering that maxillofacial surgeons encounter cancerous lesions more frequently for the diagnosis and treatment of oral cancerous lesions, this situation becomes more explanatory. However, in the responses to the question regarding the approach to a suspected case of oral cancer, oral surgeons’ high rates of incorrect responses, such as excision and biopsy of the lesion, suggest that oral surgeons demonstrate a more invasive approach to cancerous cases.

Only 21.3% of the participants in our study responded “Yes” to the question, “Do you have sufficient knowledge and experience about oral cancerous lesions?” The participants were grouped according to their responses about their knowledge and experience of oral cancers (Yes/Not Sure/No). When the responses to other questions of the survey were evaluated among the groups, the percentage of “Yes” responses to the question, “Do you have sufficient knowledge and experience about oral cancerous lesions?” varied between 27.4% and 75.4% among participants who answered the questions correctly. Among participants who answered the survey questions incorrectly, this rate ranged from 0% to 17.1%. Additionally, participants who stated that they had previously received education about oral cancers indicated that they had sufficient knowledge at a rate of 34.4%, whereas this rate was 10.9% among participants who had not received any education. These results indicate a lack of knowledge and experience among dentists participating in the survey regarding oral cancerous lesions.

The participants in our study were examined in two different subgroups based on whether they had previously diagnosed a cancerous lesion clinically, and the differences in responses to other questions between the groups were statistically analyzed. According to this, 70.8% of participants who stated that they have sufficient knowledge and experience about oral cancers had previously diagnosed a cancerous lesion. This indicates that these practitioners are more equipped to deal with cases they frequently encounter clinically. Furthermore, among those who had previously diagnosed an oral cancerous lesion, 42.5% stated that they had previously received education about oral cancers, whereas this rate was 25.4% among those who had not previously diagnosed any cancerous lesion. These results emphasize the clinical importance of oral cancer education for dentists.

When looking at similar studies conducted in Turkey before, it has been reported that dentists have gaps in their knowledge regarding oral cancers, and it has been suggested that dental education at both undergraduate and postgraduate levels should be organized considering these deficiencies [19, 20]. Our study similarly reveals the lack of knowledge among participants regarding the diagnosis and treatment of oral cancerous lesions.

Therefore, it may be appropriate to rearrange the dentistry curriculum in Turkey to increase dentists’ interest in oral cancers and thus eliminate knowledge gaps. In dentistry education, it is necessary to include the critical role played by dentists in this regard, that survival time can be significantly increased with early diagnosis of oral cancer. In addition, courses and seminars organized by dental chambers on oral cancers will be useful in order to share current information about oral cancers with dentists in the post-graduation period.

## Conclusions

In this study, the knowledge of dentists in Turkey about oral cancerous lesions was questioned. As a result, deficiencies in both clinical and theoretical knowledge were observed. It is crucial for dentists to be knowledgeable about oral cancers for early diagnosis and treatment of possible cancerous lesions. Therefore, our study highlights the necessity of increasing dentists’ knowledge about oral cancers through undergraduate education and continued education thereafter. Further studies with larger samples are needed to better identify lack of knowledge.

One of the limitations of this study is the low response rate (26.4%) received from dentists to whom the survey was sent. This low response rate limits the sample size and therefore obtaining a more generalizable result. Additionally, collecting responses to survey questions online is a limiting factor in ensuring that the questions are understood and answered correctly. In addition, the asymmetric distribution in the age ranges of the participants (79.8% of the participants were between the ages of 25-30) did not make it statistically possible to group the participants according to their age ranges. For this reason, the relationship between professional experience and the answers to the survey questions could not be examined.

### Supplementary Information

Supplementary Material 1.

## Data Availability

The datasets used or analyzed during the current study are available from the corresponding author upon reasonable request.
