# Supplementary material for: Evaluation of Turkish dentists’ knowledge about oral cancer and oral mucosal lesions
Source: BMC Oral Health. 2024 Jun 29;24:755. doi: 10.1186/s12903-024-04533-x (PMC11218260; doi:10.1186/s12903-024-04533-x)
Supplement: Supplementary file 1 — Supplementary Material 1. [file 12903_2024_4533_MOESM1_ESM.pdf]

Survey of Dentists' Knowledge About Oral Cancer And Mucosal Lesıons

Dear Participant, this research titled Dentists' Knowledge About Oral Cancer And Mucosal Lesıons was conducted by Dr. Mert Karabağ and Dr. Zeynep Gümrükçü. The research was planned to evaluate dentists' knowledge and experience about oral cancers. We work with the results obtained from your answers. Can be planned. Therefore, it is of great importance that you answer all the questions sincerely. Your participation in the research is voluntary. The information obtained through this form will remain confidential and will be used only for research purposes (or "scientific purposes"). You can choose not to participate in the study or you can stop filling out the survey if you do not want to. Do not write your name and surname on the survey form. Our survey consists of 16 questions that question your personal (subjective) opinions and objective information. Indicate your answers by ticking the appropriate option among the options below the questions. For questions where you can select more than one option, please select all the options that apply to you. If there is an "other" option among the answers to the question and your answer is not among the available options, then write your answer in the space in the other option. Thank you for answering the survey. If you have any questions about the study, you can contact:

Dr.Mert Karabağ-RTEU – Faculty Of Dentistry Oral And Maxillofacial Surgery Department

E-Mail: **Mertkarabag@Yahoo.Com** Tel: +904642220000

Survey Link: https://forms.gle/5ewWud79YfRnGi396

1. Age:

□25-30 □45-50

□30-35 □50-55

□35-40 □55+

□40-45

2. Title:

□Dentist

□Specialist

□Associate Professor

□Professor

3. Speciality (If Any)

□Oral and Maxillofacial Radiology □Prosthodontics

□Oral and Maxillofacial Surgery □Orthodontics

□Endodontics □Pediatric Dentistry

□Periodontics □Restorative Dentistry

4. Do you have sufficient knowledge and experience about oral cancerous lesions?

□Yes

□I am not sure

□No

5. Have you ever had a cancerous lesion diagnosed in your clinic?

□Yes

□No

6. Which of the following is a predisposing factor for oral cancer? (You can mark more than one answer)

□Genetic Diseases □Tobacco Smoking

□UV Sunlight □Alcohol

□Age □Mouth Breathing

□HPV □Malnutrition

□Sex

7. Which is not a pre-cancerous lesion?

□Erythroplakia □Aphthous Ulcer

□Leukoplakia □Oral Submucous Fibrosis

8. What is the most common cancer in the oral region?

□Aphthous Ulcer □Focal Epithelial Hyperplasia

□Squamous Cell Carcinoma □Verrucous Carcinoma

9. Where are oral cancers most common in the oral cavity?

□Gingiva □Tongue

□Floor of Mouth □Palate

10. Which is the clinical finding of squamous cell carcinoma?

□Gingival Recession □Tooth Sensitivity

□Red-Ulcerative Areas □Xerostomy

11. Which is the most important microscopic finding of oral cancer?

□Cellular Degeneration □Dysplasia

□Hypertrophy □ Decrease in the number of cells

12. How would you approach a case in which you suspect oral cancer, whose anamnesis states that it occurred 5 days ago?

□Biopsy □Follow-up

□Consultation to a Specialist □Total Excision

13. How long is the follow-up period for a mucosal lesion that raises suspicion of cancer?

□1 week □3 weeks

□2 weeks □4 weeks

14. Do you have sufficient knowledge and experience about oral biopsy techniques?

□Yes

□I am not sure

□No

15. In which of the following liquids should biopsy material be stored?

□Ringer's Lactate □Formalin

□Saline □Alcohol

16.Have you received any training on oral cancer before?

□Yes

□No
